# Social Information Embedded in Vocalizations Induces Neurogenomic and Behavioral Responses

**DOI:** 10.1371/journal.pone.0112905

**Published:** 2014-11-10

**Authors:** Lynda C. Lin, David R. Vanier, Sarah E. London

**Affiliations:** 1 Department of Psychology, University of Chicago, Chicago, Illinois, United States of America; 2 Institute for Mind and Biology, University of Chicago, Chicago, Illinois, United States of America; 3 Committee on Neurobiology, University of Chicago, Chicago, Illinois, United States of America; Utrecht University, Netherlands

## Abstract

Social cues facilitate relationships within communities. Zebra finches form long-term stable mate pairs and produce offspring within a multi-family, multi-generational community that can include hundreds of birds. Males use song to communicate in this complex environment. Males sing as part of their courtship display but also abundantly throughout each day, suggesting a role for their vocal signature outside of a reproductive context. One advantage of a vocal social cue is that it can be exchanged when birds are out of visual contact, as regularly occurs in a zebra finch community. Previous works have demonstrated that females hearing song are affected by their social relationship to the bird singing it, and the immediate social context. Here, we probed the question of whether or not the song itself carried social information, as would be expected from the situations when males sing outside of view of the female. We quantified behavioral and neurogenomic responses to two songs we predicted would have distinct “attractive” qualities in adult females housed in either mixed sex or female-only social communities. Our results show that only mixed sex-housed females show distinctive behavioral and neurogenomic responses to attractive songs. These data are consistent with the idea that the acoustic properties of song carry social information, and that the current social situation modulates the neural and behavioral responses to these signals.

## Introduction

Animals that live in social environments exchange a variety of cues that enable individuals to establish, maintain, and dissolve relationships. Zebra finches form long-term stable mate bonds within the large multi-family communities they live in. Song is a key component in their social communication. Only males sing. They sing as part of their courtship display but also sing abundantly outside of this context. Females do not sing but are often the recipients of these complex auditory cues.

Females use song as part of mate selection, can discriminate their mate's song from a novel song, and are typically affected by the social environment in which they hear song c.f. [1]–[11]. Visual cues can enhance mate choice, but consistent with its function as a long-distance social cue, song alone can elicit female preference behavior [12]. Song quality is clearly a factor in mate choice but unlike some other songbirds, zebra finches do not have a single acoustical trait that determines its effectiveness in mate choice scenarios [5], [13]–[16]. Notably, females tend to respond similarly to particular songs, suggesting that bout characteristics are meaningful. Females consistently prefer longer songs with high syllable diversity, consistent with the idea that song provides social information that is an honest signal of male fitness [3], [5], [8], [9], [17]–[22].

Here, we asked about the social meaningfulness of song, stripped of environmental context, and presented in standardized, controlled playback conditions. We assayed adult female responses to passive song playbacks to test two hypotheses: 1. social information embedded in the acoustic structure of song is reflected in both neural and behavioral responses to playbacks, and 2. recent social experience alters neural and behavioral responses to song playbacks. We tested two populations of females. All of the birds were raised in normal aviary conditions, but were housed in two different social communities at the time of the experiment. We measured unmanipulated behavioral responses to playbacks of two songs we predicted to have distinct attractive qualities, here termed “Popular” and “Unpopular” because their structure is associated with mate choice preferences. In the same birds, we assayed neurogenomic responsiveness to the two songs experienced in the behavioral paradigm.

This study therefore combines the sensitive and selective patterns of genomic activation initiated by hearing song in the auditory forebrain and the fact that perceptual processing is linked to behavioral response [10], [23]–[25]. Immediate early genes show differential induction in the auditory forebrain after novel and familiar song playbacks, and when a bird hears socially-related male (mates) sing [10], [26], [27]. We examined the immediate early gene Activity-regulated cytoskeleton-associated protein (Arc) because its transcriptional dynamics permit analysis of two experiences separated in time within the same brain cells [28]. Behavioral data suggest that females associate song with the singer [1]–[11]. Therefore, using passive playbacks of songs novel to all birds and with distinct structural properties, we could test the potential for song structure to signal differential neural and behavioral outcomes. Our findings are consistent with the idea that social information is embedded in the acoustic features of song.

## Methods

### Behavior

#### Subjects

Procedures were in accordance with the National Institute of Health guidelines for the care and use of animals for experimental procedures and approved by the University of Chicago Institutional Animal Care and Use Committee (ACUP #72220).

We used two Populations of adult (> Posthatch Day 90) zebra finch (*Taeniopygia guttata*) females, named here for their housing conditions. All birds were raised through adulthood in flight breeding aviaries that housed males and females of all ages. The “Mixed Sex” (MS) birds (n = 12) continued to live in breeding aviaries, in a room that contained 5 such aviaries, through the course of the experiment except when being tested. Five months prior to the start of the experiment, the “Single Sex” (SS) birds (n = 14) were removed from breeding aviaries and placed in an aviary that housed only adult females, in a room that contained only females. SS birds continued to live in this female-only social situation for the duration of the experiment except when being tested. All birds were housed on a 14∶10 light:dark cycle. Water and seed were provided *ad libitum*.

#### Behavioral Paradigm

We initially used a one-day behavioral paradigm but logistical considerations required that we switch to a two-day paradigm. All birds were run in pairs, with counterbalanced song playback order. In the one-day paradigm, females (n = 4 MS birds) were caught in their respective home aviaries and placed individually into a sound-attenuating chamber that contained a video camera, a light and speakers. Each bird was given 60 minutes to acclimate to the chamber. The birds then heard 30 minutes of song playback of one song, followed by 30 minutes of silence, then a second 30 minute playback period of the second song. Each playback period consisted of one instance of the song, with one bout repeated every 10 seconds (i.e. 6 song bouts per minute). Females were removed from the chamber 15 minutes after the second song playback period ended. We started recording the bird's behavior 10 minutes before the first song playback to capture baseline activity levels.

Birds in the two-day experiment (n = 8 MS, n = 14 SS) also had a 60 minute acclimation period and 30 minute of song playback with video recording, but playbacks were separated by 24 hours instead of 30 minutes. Birds were returned to their home aviaries between playback sessions. We again recorded the bird's behavior starting 10 minutes before song playback through the 30 minutes of song playback.

#### Song stimuli

To ensure that none of the females had previously heard stimulus songs, we chose four from a database of recordings obtained from birds at another university. We created two Song Pairs. Each pair consisted of a Popular and an Unpopular song. Criteria for “popularity” were based on reports that female zebra finches prefer long songs whose bouts (song units of several repeated elements) have a large number of different types of syllables [1], [2], [5], [8], [9]. Sound Analysis Pro (SAP2011) was used to determine song bout length and acoustic characteristics, and to count and classify syllables, excluding introductory notes [29]. We considered a syllable unique if its duration and frequency modulations were visually distinguishable from others in the bout. We assembled the song pairs based primarily on the total number and variety of syllables represented in a song.

**Figure 1:**
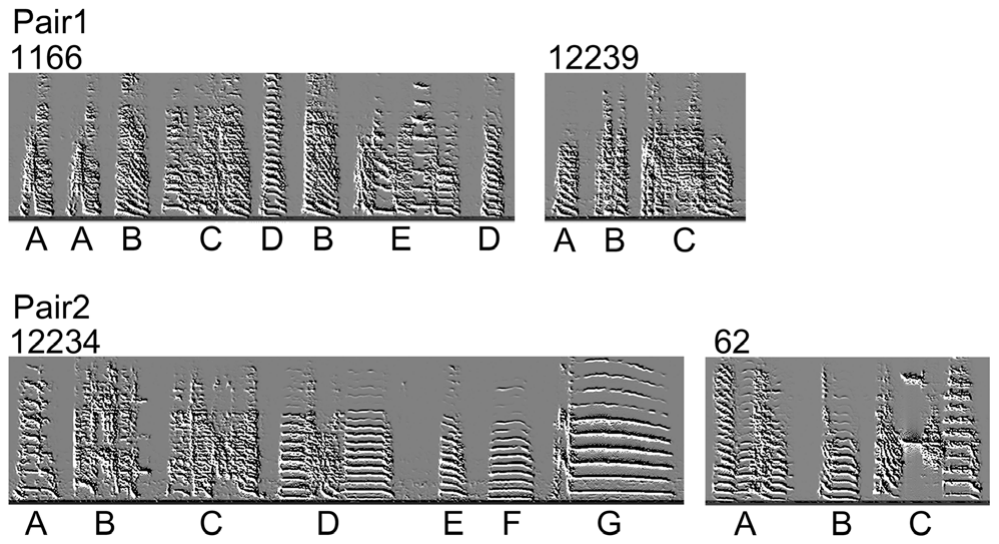
Song Pairs used for playbacks. Spectrograms of the two Popular songs (1166 and 12234) and two Unpopular songs (12239 and 62) combined into Pair 1 (1166 and 12239) and Pair 2 (12234 and 62). Capital letters designate each syllable below its position; different letters indicate unique syllables within a song.

Song Pair 1 was comprised of songs 1166 and 12239 (Figure 1). We considered 1166 to be a Popular song; it had 8 syllables, 5 of which were unique, and a 1.05 second average bout duration. 12239, the Unpopular song in Song Pair 1, had 3 syllables, all of them unique, and an average bout duration of 0.43 seconds. Song Pair 2 consisted of Popular song 12234 (7 syllables, all of them unique; average bout duration 1.47) and Unpopular song 62 (3 syllables, all of them unique; average bout duration 0.60 seconds). The Popular and Unpopular songs used had overlapping means and variances for the acoustic characteristics of amplitude, pitch, frequency modulation, amplitude modulation, entropy, and goodness of pitch (SAP2011; Table S1).

#### Behavioral scoring

We developed an ethogram to capture the behavioral profile of each bird before and during song playbacks (Table 1). We used custom-coded key strokes in JWatcher to quantitatively score and analyze these behaviors from the videos [30]. Seven different behavioral measures were scored by one person (LCL) blind to the bird's conditions. Three (preening, looking around, and eating) were analyzed for their duration because they occur continuously for long periods of time (on the order of seconds). The other four (long hops, short hops, beak wipes, and calls) were coded only for the number of times the birds performed these behaviors because their time duration was less than one second. Behaviors were scored for 10 minutes before the song started playing to capture baseline measures, and during the 30 minutes of song playback. For analysis, behavioral measures from the playback period were normalized to the pre-playback period to account for individual differences in general activity that does not reflect a change from the song experience.

**Table 1:** Ethogram for quantitative behavioral analysis.

Code	Behavior	Description
b	beak wipe	bird wipes beak on perch or other surface
e	eat or drink	bird eats or drinks, can be floor or food dish
g	go	song playback starts
h	long hop	hop from perch to floor, side of cage to food dish, food dish to perch, etc
l	look around	side-to-side head movements but no body movement
p	preen	bird fluffs, shakes, scratches, grooms with beak
q	quit	quit for states: eat, preen & look around
s	small hop	hop along perch or local hop (<1″) or spin-turns
c	call	vocalization of an individual, short note

In addition to scoring the seven behaviors, we also coded the time of delay to each bird's first behavior. Delay was defined as the duration between song playback onset and the bird's first behavior, which could be any of the behaviors with the exception of look around. This definition of delay, along with the decision to exclude look around as one of the behaviors that would determine the end of the delay, is similar to what Stripling and colleagues (2003) called “response latency” in their song discrimination study.

### Cellular compartment analysis of temporal activity by fluorescence in situ hybridization (catFISH)

#### Acute song playback

To test the auditory forebrain patterns of the immediate early gene Arc induction in response to the experimental songs without confounds of familiarity, we waited to do song playbacks for catFISH for at least 2 weeks after birds were tested for behavioral responses.

For catFISH playbacks, females were removed from their home aviaries and placed individually into sound-attenuating chambers overnight for ∼16 hours. Each female was played one song for 15 minutes, followed by 10 minutes of silence and then 5 minutes of the second song. Song playback periods consisted of a single song bout, repeated once every 10 seconds. Each female heard the same Song Pair and Popular-Unpopular song order as they had in the behavioral experiment. Immediately after the second song finished, females were removed from the chambers and the whole brain was rapidly dissected and flash frozen. Brains were stored at -80°C until use.

#### Fluorescent in situ hybridization (FISH)

We collected 8 µm parasagittal sections from the auditory forebrain of experimental birds plus three adult females placed into chambers without song playback as silence negative controls (n = 3). All sections were from the left hemisphere. Auditory forebrain sections 100–300 µm from the midline were processed for FISH [31]. We also collected 3 sections from the auditory midbrain (nucleus mesencephalicus lateralis pars dorsalis; MLd) of all of these birds to assay intensity of auditory experience [10].

We hybridized with both Arc and ZENK (*zif268, egr-1, ngfi-a, knox24*) antisense riboprobes. In the auditory forebrain, ZENK dual label was used as visual aid to cell morphology only. In the MLd, we used it as the measure of auditory experience, as ZENK activation positively correlates with hearing a stimulus, regardless of its novelty or quality [10]. To construct the Arc plasmid probe template, we PCR amplified a 653 bp fragment of zebra finch Arc cDNA (AS primer: TGGAAGAAGTCCATCAAGGC; S primer: TTGCGCCAGAGGAACTGGTC, GenBank Accession # EF076776; [32]) from whole brain cDNA reverse transcribed with random hexamer primers from total RNA (Trizol, per manufacture's protocol, Life Technologies). The Arc amplicon was ligated into PCRScript (Agilent Technologies) per manufacturer's protocol. We sequenced the plasmid to confirm the insert. The ZENK clone is as described in [33]. Plasmids were linearized to use as in vitro transcription templates for DIG- or biotin- labeled riboprobes from the Arc or ZENK clone, respectively. Probes were purified before use (RNeasy, Qiagen).

For Arc catFISH with ZENK double-label, sections were dried at room temperature, fixed for 7 min in 3% paraformaldehyde (pH 7.4), rinsed 4x in 0.025 M KPBS (pH 7.4) equilibrated in 0.1 M TEA for 3 min, then treated with 0. 25% v/v acetic anhydride in TEA for 10 min. Sections were washed 2x in 2X SSC, then dehydrated serially with ethanol. After drying at room temperature, sections were hybridized 16 hr at 65°C hybridization solution (50% formamide, 2× SSPE [pH 7.4], 2 mg/ml tRNA, 1 mg/ml bovine serum albumin, 300 ng/ml polyadenylic acid, 0.1 M DTT) containing 400 ng each of Arc and ZENK riboprobes. After hybridization, sections were rinsed in 2X SSC to remove coverslips and washed in 0.1X SSC/50% formamide and 0.1X SSC, all at 65°C. Sections were then rinsed in TN (100 mM Tris pH 7.5, 150 mM NaCl), and treated with 0.6% H_2_O_2_ in 2X SSC for 30 min at room temperature to exhaust endogenous peroxidases. After rinsing in TN, sections were blocked 1 hr at room temperature, then incubated 2 hr at room temperature with a 1∶100 dilution of POD-conjugated anti-DIG primary antibody in block solution. Slides were washed in TN before application of Tyramide Signal Amplification (TSA; Life Technologies) with a 594 nm fluor for 1 hr at room temperature. Slides were then washed in TN and treated with 0.6% H_2_O_2_ to exhaust residual peroxidases from the TSA procedure. Sections were washed in TN, incubated for 2 hr at room temperature in a 1∶100 dilution of POD-conjugated anti-biotin primary antibody in block solution, washed in TN, then incubated 1 hour at room temperature in TSA with a 488 nm fluor. Slides were washed in TN, treated with 1∶10000 dilution of DAPI in TN for 2 min, and washed again before coverslipping in aqueous mounting media.

#### Image capture

**Figure 2:**
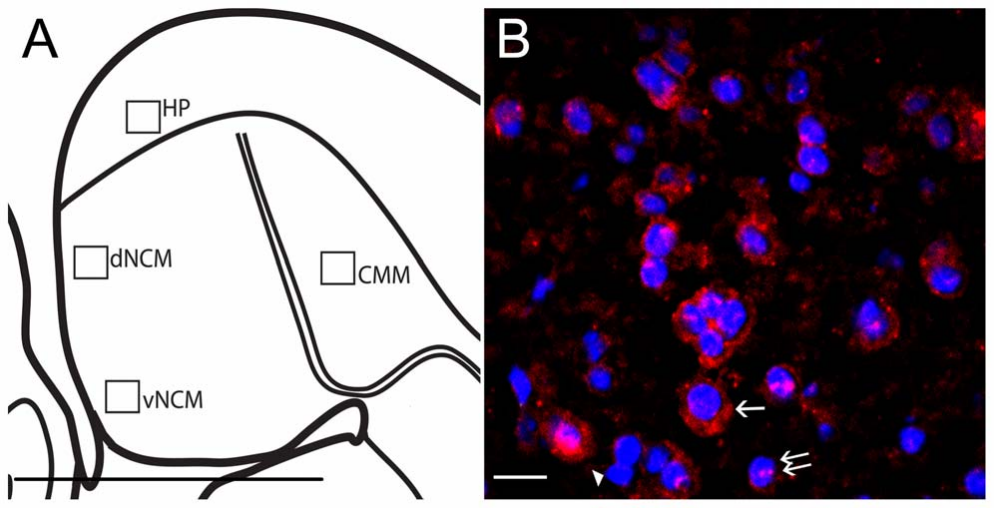
catFISH in auditory forebrain. A) Schematic of auditory forebrain in sagittal plane. Boxes represent brain regions imaged and measured from catFISH-processed brain sections. Anterior is toward the right, dorsal is up. HP  =  hippocampus, CMM  =  caudomedial mesopallium, dNCM  =  dorsal caudomedial nidopallium, vNCM  =  ventral caudomedial nidopallium. B) 60X image of catFISH procedure showing Arc mRNA (red) and DAPI-stained nuclei (blue). Single arrow points to cytoplasmic-only labeled cell, double arrow points to double-localized cell, and arrowhead indicates cell without Arc mRNA staining. Scale bar in A = 1 mm, B = 10 µm.

We captured three regions of auditory forebrain, caudomedial mesopallium (CMM), dorsal caudomedial nidopallium (dNCM), ventral NCM (vNCM), and an adjacent area of hippocampus (HP) as a within-section staining intensity control (Figure 2). We also imaged a central portion of MLd. Sections were imaged in 2 µm Z stacks with a 60X objective on a Olympus DSU spinning disk confocal microscope (Olympus Corporation of the Americas, Center Valley, PA) with an Evolve EM-CCD camera (Photometrics, Tucson, AZ) run by SlideBook v5.0 software (Intelligent Imaging Innovations, Denver, CO). Exposure times for Arc and ZENK staining were set to be equivalent for all slides after scanning through sections within a set, capturing test images, and examining the visual result as well as the intensity histograms. DAPI exposure was taken with the auto exposure function independently for each slide. Images were deconvolved in SlideBook with a subtraction constant of 0.9 before analysis.

#### mRNA quantification

On catFISH-processed sections, we manually counted four measures in each auditory forebrain image: total number of cells (DAPI), intranuclear Arc staining, cytoplasmic Arc staining, and double-localized (intranuclear and cytoplasmic) Arc staining. Cell counting was performed blind to the condition of the birds (by SEL). We used the ZENK channel on images to aid identification of cell morphology but because different immediate early genes are not always induced together, all auditory forebrain in situ hybridization quantification was based on Arc label [32], [34], [35]. We normalized Arc signal counts first by the total number of cells captured in the image; this proportion was then divided by that same measure from the HP. In the MLd, we quantified the intensity measures of the ZENK staining within MLd after normalizing to the level of staining in the adjacent midbrain for each image (ImageJ 1.45 s).

#### Ovarian morphology

To assess the ovarian status of the birds, we measured the number and size of follicles at the time of catFISH tissue collection (typically 2 weeks after behavioral tests). Follicles were categorized as medium, small, or very small based on size and presence or absence of yolk (medium: 2–4 mm and yolk present, small: 1–2 mm and no yolk present, very small: 0.1–1 mm and no yolk present). We originally reserved the “large” class for even larger yolk-filled follicles but none were observed.

#### Statistical Analysis

We used one-way analysis of variance (ANOVA) to examine Paradigm and Song Pair effects (SAS software, SAS Institute Inc., Cary, NC). To compare individual normalized behavioral responses, behavioral rates, and delay times between Song Types (Popular, Unpopular), and Populations (MS, SS), and the interaction between these variables, we used two-way ANOVA (SAS software version 9.4); Population differences based on Song Type were analyzed with one-way ANOVA. Delay times were non-normally distributed based on Shapiro-Wilk's analysis; we therefore log transformed these data prior to ANOVA testing. We also used multivariate ANOVA (MANOVA) statistics to analyze behavioral measures collectively across Populations (SAS software version 9.4). Normalized cell proportions for each auditory forebrain region were analyzed with two-way ANOVA (SAS software). For MLd, ZENK intensity measures were compared directly for Song Type with one-way ANOVA (SAS software version 9.4). We used one-way ANOVA to test for differences in the abundance of ovarian follicle categories between MS and SS females, and linear regression to test for relationships between ovarian follicle status, Delay times, and catFISH results (SAS software version 9.4).

## Results

### Behavioral Responses to Song Playbacks

#### Paradigm and Song Pair did not affect behavioral measures or delay times

Testing birds on one day or across two days did not significantly affect any behavioral measure (beak wipe: F(1,25) = 0.14, p = 0.72; eating: F(1,25) = 0.65, p = 0.43; long hop: F(1,25) = 1.16, p = 0.29; look around: F(1,25) = 3.03, p = 0.10; preening: F(1,25) = 3.48, p = 0.09; short hop: F(1,25) = 2.75, p = 0.11; calls: F(1,25) = 0.31, p = 0.58) or delay times (F(1,25) = 2.08, p = 0.16). We therefore combined data from the two paradigms for all other analyses.

We also compared the behavioral responses and delay times between Song Pairs. We found no differences in behavioral responses (beak wipe: F(1,25) = 0.21, p = 0.65; eating: F(1, 25) = 0.01, p = 0.97; long hop: F(1, 25) = 2.95, p = 0.09; look around: F(1, 25) = 0.13, p = 0.71; preening: F(1, 25) = 0.92, p = 0.34; short hop: F(1, 25) = 3.00, p = 0.10; calls: F(1,25) = 0.54, p = 0.47) or delay durations (F(1, 25) = 0.08, p = 0.78). We therefore only considered the category of Popular and Unpopular in further analyses.

#### MS females show distinctive delay duration for Popular song playbacks

We tested for main effects of Population and Song Type, and the Population * Song Type interaction for behaviors in the ethogram. We found no main effects of Population (beak wipe: F(1, 25) = 2.10, p = 0.16; eating: F(1, 25) = 0.71, p = 0.41; long hop: F(1, 25) = 1.90, p = 0.18; look around: F(1, 25) = 0.97, p = 0.34; preening: F(1, 25) = 1.32, p = 0.26; short hop: F(1, 25) = 2.72, p = 0.12; calls: F(1,25) = 3.69, p = 0.07) or Song Type (beak wipe: F(1, 25) = 0.38, p = 0.54; eating: F(1, 25) = 0.02, p = 0.89; long hop: F(1, 25) = 1.37, p = 0.25; look around: F(1, 25) = 0.09, p = 0.77; preening: F(1, 25) = 1.72, p = 0.20; short hop: F(1, 25) = 3.61, p = 0.07; calls: F(1,25) = 1.29, p = 0.27), and no significant interaction (beak wipe: F(1, 51) = 3.45, p = 0.08; eating: F(1,51) = 2.46, p = 0.13; long hop: F(1,51) = 1.49, p = 0.23; look around: F(1, 51) = 0.23, p = 0.64; preening: F(1, 51) = 1.47, p = 0.24; short hop: F(1,51) = 2.01, p = 0.17; calls: F(1,51) = 0.01, p = 0.91) for any behavior.

**Figure 3:**
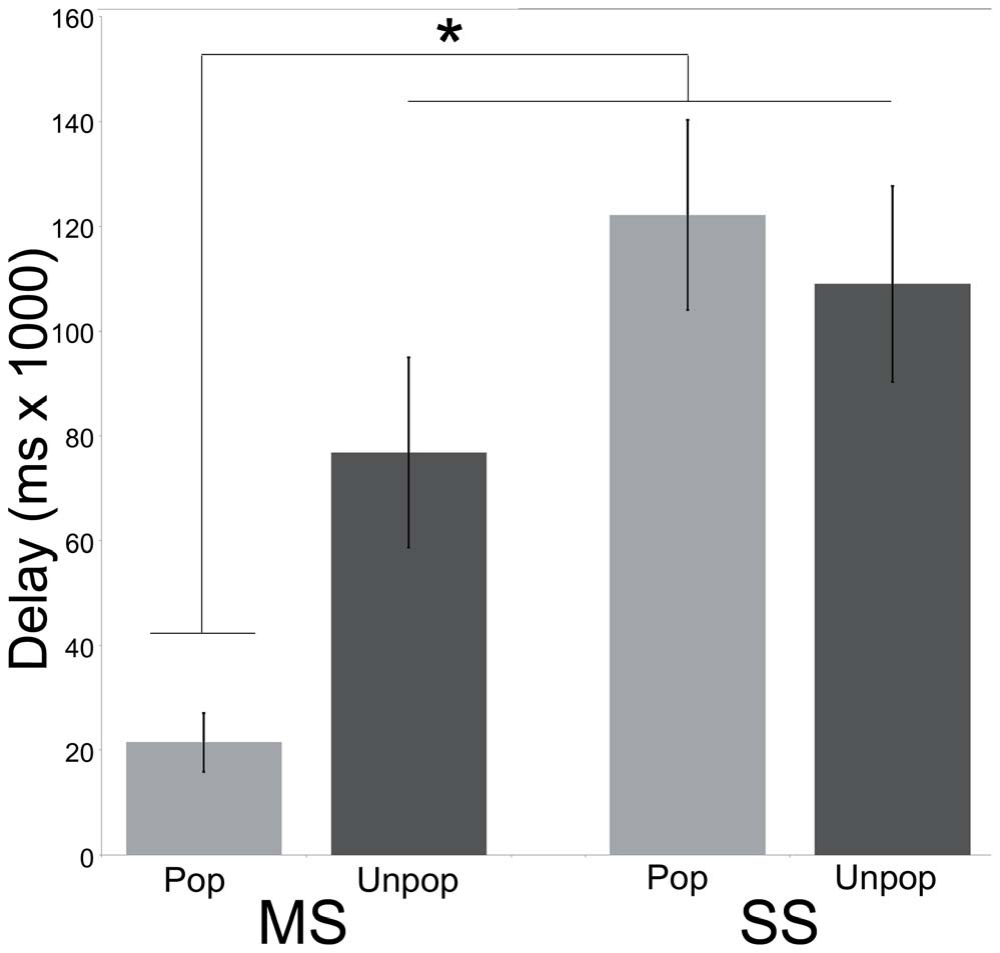
Popular song playback induces selective behavioral response. MS birds display significantly shorter delay times when they hear Popular compared to Unpopular song playbacks. MS delay times during Unpopular song playbacks are no different than SS birds when they hear either Popular or Unpopular song playbacks. Error bars indicate s.e.m.

Based on precedence for a “quiet listening period,” we also measured the time delay from song playback onset to the first, non-look around behavior [36]. Delay duration did show a significant main effect of Population (F(1,24) = 6.57, p<0.01) and a significant Population*Song Type interaction (F(1, 51) = 11.60, p<0.01), but no main effect of Song Type (F(1,24) = 1.14, p = 0.23). SS females show no difference in delay times when presented with playbacks of Popular or Unpopular songs and MS females show the same length of delay when presented with Unpopular song playbacks. MS females, however, show a significantly shorter delay time (F(1,11) = 8.77, p<0.01) when they hear Popular compared to Unpopular song playbacks; one-way ANOVA with Tukey post-hoc tests confirm that Delay time for the MS birds after hearing Popular song playbacks is significantly different from the other conditions (F(3,48) = 6.60, p<0.01) (Figure 3).

#### Baseline behavior does not predict time of delay

We noted that the MS females had higher overall baseline levels of activity than SS birds prior to song playback onset (F(7,44) = 3.79, p = 0.05, Wilk's λ = 0.66). It was therefore possible that a bird's behavior prior to song onset reliably predicted the delay time, which would suggest that delay times reflect general activity levels or states, not behavioral responses to song playback per se. If so, it would be reasonable to expect that higher behavioral rates would correlate with shorter delay times (i.e. more active birds sit quietly for less time during playbacks). Linear regression of the baseline behavioral measures to delay times, however, failed to find a significant overall relationship (F(7,44) = 2.29; p = 0.08), had low predictive power (R^2^ = 0.19), and showed a positive correlation, opposite to the expected negative correlation.

#### MS and SS birds perform behaviors at same rate during song playback period

Because of the significantly shorter delay times for the MS birds, they had a longer time period to display the other behaviors after the onset of Popular song playback compared to all other conditions. MS birds did have overall higher levels of activity than SS birds during song playbacks (F(7,44) = 2.41, p = 0.04, Wilk's λ = 0.72). Therefore, we considered the possibility that rates of activity were different once the birds began to behave after the delay was over. Rate differences might indicate some persistent alteration in behavioral motivation after the delay ended, distinct from simply the number and duration of behaviors we previously analyzed. When we analyzed the rates of each behavior for main effects of Population or Song Type, and their interaction, we detected only one significant difference: the rate of preening was higher in MS compared to SS birds (F(1,25) = 6.29, p = 0.02). There is a similar Population difference in preening in the baseline data (F(1,25) = 6.46, p = 0.02), that disappears in the normalized data (F(1,25) = 1.32, p = 0.26). Further, there is no effect of Song Type (F(1,25) = 0.39, p = 0.53) or a Population*Song Type interaction (F(1,51) = 0.01, p = 0.93) on preening rate, indicating that behavioral rates, including preening, are not altered by song playback.

### Genomic Responses to Song Playbacks

#### Song Pair did not affect Arc induction

As expected, we found a significant difference between labeling intensity in birds left in silence compared to other birds (F(1,28) = 5.20, p<0.01) using MLd ZENK staining to assay the magnitude of auditory experience. There was no difference in MLd staining between birds that heard a Popular or Unpopular song first (F(1,25) = 2.08, p = 0.17), or between MS and SS birds (F(1,25) = 1.19, p = 0.29). We analyzed three categories of Arc hybridization: cytoplasmic only (Arc induced by first song playback only), nuclear only (Arc induced by second song playback only), cytoplasmic and nuclear double-localized (Arc induced by both songs), in three subregions of auditory forebrain: CMM, dNCM, and vNCM. All measures were significantly lower in Silence birds compared to birds who heard song playbacks (F(1,28)≥2.68, p≤0.03). Further, in the females that experienced song playbacks, there were no differences in any measure between the Popular songs (F(1,25)≤1.61, p≥0.13) or Unpopular songs (F(1,25)≤0.97, p≥0.33) from Song Pairs 1 and 2. We therefore combined data from both Song Pairs to analyze subcellular patterns of Arc label in relation to Population and Song Type. We report results from birds that heard playbacks below.

#### MS females have a distinctive Arc response to Popular song playbacks

**Figure 4:**
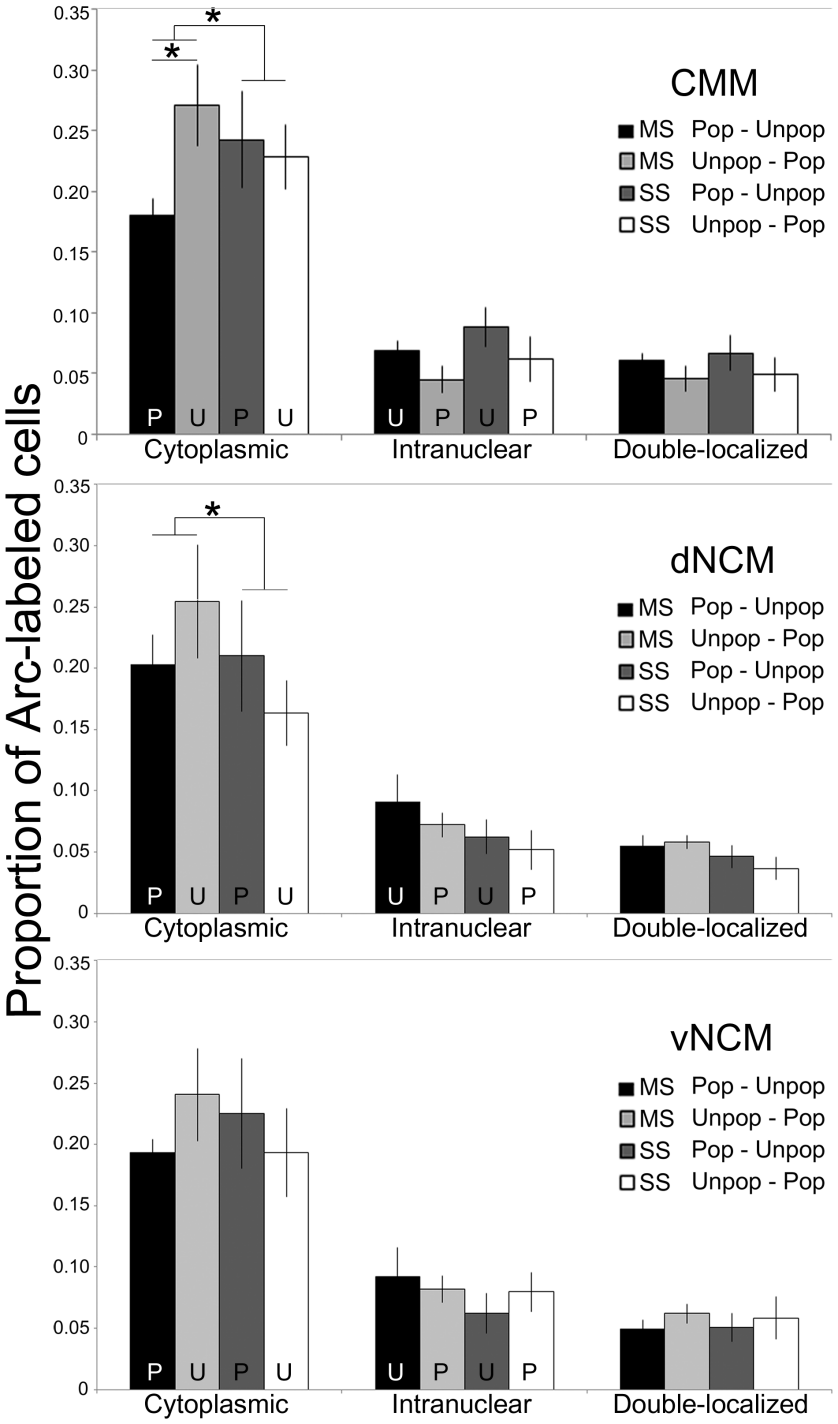
catFISH results from CMM, dNCM, and vNCM. Bars show the normalized proportion of cells with cytoplasmic, intranuclear, or double-localized Arc mRNA. For clarity, “U” and “P” indicate that either Unpopular or Popular song playbacks induced the cytoplasmic and intranuclear staining in the four conditions. Asterisks denote significant (p<0.05) differences. Error bars indicate s.e.m.

In CMM, there was a significant interaction between Population and Song Type for CMM cytoplasmic (F(1,51) = 8.47, p<0.01) staining, with similar trends in intranuclear (F(1,51) = 3.50, p = 0.06) and double-localized (F(1,51) = 3.84, p = 0.06) staining. The Population* Song Type interaction in cytoplasmic staining is due to fewer Arc labeled cells in the MS birds who heard Popular song first compared to MS birds that heard Unpopular song playbacks first (F(1,11) = 5.32, p = 0.04); i.e. there is lower Arc expression in CMM in response to Popular song playbacks. A similar trend is seen in the MS intranuclear label (F(1,11) = 3.47, p = 0.06). SS birds show no difference in cytoplasmic or intranuclear cell numbers after Popular and Unpopular playbacks (cytoplasmic: F(1,13) = 0.06, p = 0.81; intranuclear: F(1, 13) = 0.05, p = 0.86) (Figure 4).

In NCM, there was a significant interaction between Population and Song Type for dNCM cytoplasmic label (F(1,51) = 4.62, p = 0.03), but not for any of the other measures (intranuclear F(1,51) = 0.06, p = 0.81; double-localized F(1.51) = 0.68, p = 0.41; Figure 4). We found no significant differences in vNCM Arc labeling, though the general pattern is similar as measured in CMM and dNCM (cytoplasmic F(1,51) = 2.35, p = 0.12; intranuclear F(1,51) = 0.64, p = 0.42, double-localized F(1,51) = 0.06, p = 0.80; Figure 4).

In CMM, dNCM, and vNCM, we counted more cells with cytoplasmic Arc label than intranuclear label (Figure 4). Most cells that contained intranuclear Arc mRNA also had cytoplasmic staining, as seen in the double-localized data (Ranges for CMM: 73–91%; dNCM: 73–95%; vNCM: 65–88%; Figure 4), with no significant differences based on Song Type or Population. Additionally, no catFISH measure significantly correlated with an individual's delay time (R^2^<0.04, F(1,24)<1.20, p>0.29).

#### MS and SS birds did not differ in ovarian status

It was possible that the MS birds were in mate pairs and in an active reproductive state, while SS birds could not be reproducing. As an indirect measure of reproductive status, and therefore circulating hormone levels, we quantified three categories of ovarian follicles [37], [38]. We found no difference between MS and SS birds in medium (F(1,23) = 0.65, p = 0.43), small (F(1,23) = 2.58, p = 0.12) or very small (F(1,23) = 0.12, p = 0.73) follicles. Further, individual ovarian status did not significantly correlate with any catFISH measures (R^2^<0.003; F(1,24)<1.064; p>0.31) or with delay durations (Popular: R^2^ = 0.003, F(1,24) = 0.08, p = 0.78; Unpopular: R^2^ = 0.022, F(1,24) = 0. = 53, p = 0.47).

## Discussion

Zebra finches form long-term mate pairs and live in complex social communities. Song likely contributes to the establishment and maintenance of social bonds, including those between partners. Adult females distinguish their mate's song from other songs and social context enhances genomic responses to song playback [3], [6], [7], [10]. One advantage of an auditory signal is that it can be transmitted even when the bird singing is out of sight. Indeed, song contributes to female mate choice and is associated with male fitness [5], [9]. We therefore hypothesized that social content is embedded in song itself, independent of a specific social bond or context. We further tested if evaluation of social content relies on recent experience. Using a standardized song playback paradigm that removed environmental context, we found that only the females currently housed in a normal social environment displayed differential behavioral and genomic responses when hearing Popular or Unpopular song playbacks.

Behaviorally, the time of delay is selectively modulated in the MS birds by acoustic properties in the songs. There are two main possible explanations for these findings. One is that the MS birds have a short delay during Popular song playbacks because they initiate an active response to an “attractive” song. There was, however, no strong evidence for an increased activity level in the MS birds during Popular song playbacks. Another possible explanation is that the delay represents a quiet listening period for auditory processing or learning, and the MS birds need less time for these processes when they hear Popular songs. The delay time was previously described in a paradigm of familiar and novel song playbacks [36]. There, adult zebra finches sat quietly longer for novel song playbacks than familiar songs. This “latency” was interpreted as a quiet listening period that reflected a nonassociative song recognition learning process [36]. Our songs were initially novel to all birds, and playbacks were one-sixth the duration used in the recognition procedure; it is not clear how much learning occurred. If Popular songs were more salient, however, they could more rapidly or effectively modulate attention for processing or learning; attention has been previously proposed as an underlying mechanism for more active responses to complex songs [8]. Perhaps recent exposure to songs facilitates this process, so that MS but not SS birds make the behavioral discrimination faster during the 30 minute playbacks. Future experiments could be performed to evaluate this idea.

The MS birds also showed a selective, distinctive pattern of Arc induction in the auditory forebrain in response to Popular song playbacks. Generally, Popular song playbacks induced fewer cells to transcribe Arc than Unpopular song playbacks in MS but not SS birds; the interaction was significant in CMM and dNCM, but not vNCM, though Arc induction patterns were similar in all three regions. The effect of Song Type was more pronounced when we analyzed the cytoplasmic label compared to intranuclear and double-localized staining. The difference may reflect a difference in our detection sensitivity, as the trend is similar for both subcellular locations but we counted a lower percentage of labeled cells in the nucleus. A high percentage of cells with intranuclear Arc mRNA also contained cytoplasmic staining, suggesting that both songs activate transcription in a similar subset of cells.

Arc is required for long-term memory processes and functions as a structural protein that directs morphological substrates of synaptic strength c.f. [39]–[42]. Based on patterns of induction of other immediate early genes such as ZENK and the fact that Arc accumulates in highly activated brain areas, we would have predicted that Popular song playbacks would induce more Arc transcription than Unpopular playbacks [10], [40], [43], [44]. On the contrary, our data show that in the auditory forebrain of MS birds, hearing Popular song triggered the least potential for Arc-mediated synaptic remodeling of all of the groups. Arc is targeted to synapses to prevent synaptic strengthening in neurons that are firing, as would occur in the auditory forebrain during song playbacks [45], [46]. It seems that in MS birds, synapses are protected from Arc-mediated scaling in response to Popular songs, perhaps because of their regular contact with song in their mixed sex community. Interestingly, this effect was most pronounced in the CMM compared to NCM, where it has been proposed that the social content of song is processed [10].

As the Mixed Sex housing condition is the normal environment, our results suggest that SS birds lose discrimination between the Popular and Unpopular song on both the neurogenomic and behavioral levels. We considered that age or endocrine status could contribute to these results. All of the birds were adult females of unknown exact ages. However, it is possible, given the logistics of setting up the aviaries, that the SS population was 6–9 months older than the MS birds. Behavioral effects of aging have not been described in these birds, but it is formally possible that base energy levels or auditory discrimination capabilities were lower in the SS birds compared to the MS birds. MS birds did perform more behaviors than SS birds overall but the fact that Unpopular song playbacks elicited the same delay times in MS as SS birds demonstrates that MS birds were capable of sitting quietly for long periods. Further, several lines of evidence described above show that the delay durations were independent of overall activity levels. Although we did not directly test it, we are also not aware of specific reports of a significant age-related decline in auditory processing within adult songbirds.

It was also possible that MS and SS females differed in their estradiol levels, affecting auditory processing or behavioral responsiveness [8], [47], [48]. We surmised that it was more likely that MS females would have large mature ovarian follicles, and therefore higher levels of circulating estradiol, than SS females since they had the opportunity to breed. Ovarian-derived steroids likely do not explain our results. We found no difference between follicles between MS and SS birds, although we had to measure them weeks after the behavioral testing to avoid song familiarity confounds. However, zebra finch brains, including the auditory forebrain, synthesize estrogen and other steroids de novo that can modulate auditory processing [49]–[52]. We cannot rule out that locally-synthesized estrogen contributed to the distinctive responses of MS birds, though it is not obvious what would regulate its production differentially in MS birds during Popular song playbacks compared to the other conditions [51], [52].

Implicit in our discussion is the idea that neurogenomic indicators of song playback processing in the auditory forebrain are directly related to how a bird behaviorally responds. It is possible that Arc induction within subregions of the auditory forebrain reflect processing that informs the neural systems that control the time of delay to an active behavioral response. It is also possible that both the Arc induction and time of delay result from some as-yet-identified third process. Our experimental design permitted us to identify this brain-behavior connection but not to reconcile between mechanistic options.

This study does demonstrate that social information embedded within the acoustic structure of song is sufficient to cause selective neurogenomic and behavioral responses, but only in females who are living in normal social environments. Our stimulus songs overlapped in acoustic features, thus it is still unclear what components of the song structure might provide the signals that induce distinct brain and behavioral responses. These results, however, strengthen the connections between natural experience, neurogenomic regulation, and behavioral response.

## Supporting Information

Table S1 **Popular and Unpopular acoustic traits.**
(PDF)
