# Supplementary material for: Social Information Embedded in Vocalizations Induces Neurogenomic and Behavioral Responses
Source: PLoS One. 2014 Nov 10;9(11):e112905. doi: 10.1371/journal.pone.0112905 (PMC4226578; doi:10.1371/journal.pone.0112905)
Supplement: Table S1 — Popular and Unpopular acoustic traits. (PDF) [file pone.0112905.s001.pdf]

Table S1. Popular and Unpopular acoustic traits.

	Mean							Variance					
	Amplitude (dB)	Pitch (Hz)	FM (deg)	AM^2 (1/ms)	Entropy	Pitch, goodness	Frequency (Hz)	Pitch (Hz)	FM (deg)	Entropy	Pitch, goodness	Frequency (Hz)	AM
**Popular**	47.9±5.6	1029±74	38.3±6.6	-0.00098±0.00048	-1.71±0.36	230.85±33.7	2811.5±679	1148000±644000	648±52	0.545±0.13	37900±7600	342000±240000	0.005885±0.00135
**Unpopular**	51.25±6.3	1254±540	40.15±5.5	-0.001055±0.00035	-2.175±0.07	233±162.2	3318.5±111	2084500±45000	622.5±217	1.28±1.68	41200±59000	539000±56000	0.006395±0.00165

Values are means ± range for the two Popular and Unpopular songs.
